# Recent Progress in Understanding and Predicting Atlantic Decadal Climate Variability

**DOI:** 10.1007/s40641-017-0064-z

**Published:** 2017-04-18

**Authors:** S. G. Yeager, J. I. Robson

**Affiliations:** 1 National Center for Atmospheric Research, Boulder, CO USA; 2 National Centre for Atmospheric Science, University of Reading, Reading, UK

**Keywords:** Decadal prediction, Climate prediction, Atlantic multi-decadal variability, Thermohaline circulation, AMOC, Subpolar gyre

## Abstract

**Purpose of Review:**

Recent Atlantic climate prediction studies are an exciting new contribution to an extensive body of research on Atlantic decadal variability and predictability that has long emphasized the unique role of the Atlantic Ocean in modulating the surface climate. We present a survey of the foundations and frontiers in our understanding of Atlantic variability mechanisms, the role of the Atlantic Meridional Overturning Circulation (AMOC), and our present capacity for putting that understanding into practice in actual climate prediction systems.

**Recent Findings:**

The AMOC—or more precisely, the buoyancy-forced thermohaline circulation (THC) that encompasses both overturning and gyre circulations—appears to underpin decadal timescale prediction skill in the subpolar North Atlantic in retrospective forecasts. Skill in predicting more wide-ranging climate variations, including those over land, is more limited, but there are indications this could improve with more advanced models.

**Summary:**

Preliminary successes in the field of initialized Atlantic climate prediction confirm the climate relevance of low-frequency Atlantic Ocean dynamics and suggest that useful decadal climate prediction is a realizable goal.

## Introduction

The Atlantic is a region characterized by pronounced fluctuations in climate from one decade to the next. The term Atlantic Multi-decadal Oscillation (AMO) was coined to describe the roughly 70-year swings in a variety of instrumental and proxy records from the Atlantic region that apparently reflect a mode of natural variability of the climate system [1–3]. In a seminal early study, Bjerknes interpreted observed decadal surface temperature fluctuations in the North Atlantic in terms of a coupled atmosphere-ocean oscillation with the ocean (atmosphere) playing a driving (damping) role [4]. Subsequent examination of longer, gridded observational data sets revealed coherent patterns of decadal sea surface temperature (SST) and sea level pressure (SLP) variability in the Atlantic that were consistent with Bjerknes’ ideas, bolstering the hypothesis that low-frequency (i.e., decadal to multi-decadal) Atlantic SST anomalies were proximately caused by ocean dynamical changes [5, 6].

Analysis of early coupled global circulation models (CGCMs) revealed that intrinsic, low-frequency SST, surface air temperature (SAT), and SLP variations in the North Atlantic and Arctic were consistently associated with variations in the strength of the Atlantic Meridional Overturning Circulation (AMOC), and in particular its slow (buoyancy-forced) thermohaline circulation (THC) component [7–9]. While AMOC/THC is considered the dominant oceanic phenomenon influencing Atlantic climate, the North Atlantic Oscillation (NAO) is the dominant mode of atmospheric variability in this region [10]. Strong, positive winter NAO conditions generate a fast, tripolar North Atlantic SST response, with maximum heat loss occurring over the Labrador Sea [11–13]. NAO forcing also modulates abyssal water mass properties and induces a delayed response of the Atlantic Ocean gyre and overturning circulations [14–16] with the potential for the ocean to feedback onto the atmosphere and give rise to associated coupled modes of Atlantic variability [12]. Recent research has underscored the significance of persistent NAO buoyancy forcing in giving rise to large, low-frequency Atlantic THC variations that can influence extratropical upper ocean heat content, SST, and pan-Atlantic surface climate [17–19, 20•].

While there are many outstanding questions regarding the physical mechanisms that contribute to the observed AMO (or AMV, to reflect the more modern emphasis on broad-spectrum “variability” over “oscillation”), which we expand upon below, a number of studies have identified significant climate impacts associated with North Atlantic SST variability that have helped to define the targets for Atlantic-focused climate prediction efforts. The twentieth century AMV has been linked to observed variability in (see also references within these recent papers): seasonal climate (SAT and precipitation) over North America and Europe [21–23]; rainfall over India, northeast Brazil, and the Sahel [24, 25]; Atlantic hurricane activity [24–26]; North Atlantic atmospheric blocking frequency [27]; sea level along the east coast of the USA [28•]; high-latitude heat fluxes [29•]; and the NAO [30•, 31•]. Proxy records are also crucial for characterizing AMV and its impacts over multiple cycles prior to the instrumental record. A reconstruction derived from tree rings underscores the connectivity between low-frequency Atlantic SST and pan-Atlantic terrestrial climate and suggests significant spectral energy in a wide temporal band between about 40–128 years [32]. Greenland ice cores also indicate the presence of a strong 20-year periodicity that has been interpreted as a signature of AMV [33]. Many of these observed AMV linkages have also been found in CGCMs, either in long, unforced simulations or in sensitivity experiments [24, 34, 35, 36•]. Models have also pointed to other important impacts associated with AMOC and AMV that cannot be easily discerned from limited observations, such as the modulation of Arctic sea ice extent and thickness [37•] and Northern Hemisphere extratropical SAT and SLP [20•]. An observation-based link between multi-decadal Arctic sea ice variability and AMV has recently been established using a combination of historical records and high-resolution proxy data [38]. Although AMV has historically connoted a coherent pan-Atlantic mode of internal variability, there are other noteworthy impacts associated with potentially predictable regional changes in heat content and SST in the subpolar North Atlantic. These include large decadal shifts in ocean ecosystems [39], rates of Greenland ice melt [40•], and rates of North Atlantic carbon uptake [41].

The study of the AMV, AMOC/THC, NAO, and their inter-connections has flourished in recent decades with a diverse set of observations and modeling tools brought to bear (see [42, 43•] for useful reviews). The mechanistic understanding of low-frequency Atlantic climate variability has greatly influenced the expectations for, and interpretations of, practical prediction efforts. A review of the state of Atlantic predictability science as of a decade ago emphasized the lagged relationships between the NAO, Labrador Sea Water (LSW) formation, THC/AMOC, and decadal SST—with some indications of a feedback of SST onto the atmosphere to close the loop [44]. This causal chain continues to be a dominant conceptual paradigm that has guided much of the recent progress in Atlantic decadal prediction. However, recent studies have challenged the prevailing view that ocean circulation variability plays a fundamental role in Atlantic decadal variability and predictability [45, 46]. There is now an ongoing debate concerning the relative importance of local thermodynamical versus large-scale dynamical ocean physics for explaining low-frequency variations in Atlantic climate.

The identification of societally relevant climate impacts associated with North Atlantic ocean variability (and in particular, the upper ocean heat content and SST variability) has given impetus and focus to Atlantic decadal climate prediction work. The first pioneering papers that demonstrated the potential for skillful retrospective prediction of observed Atlantic decadal climate change using suitably initialized CGCMs appeared less than a decade ago [47–49]. However, as we have tried to highlight, the intellectual lineage of these results can be traced through at least a half-century’s worth of observational and modeling work that has elucidated the nature of low-frequency Atlantic climate variability. Current Atlantic climate prediction research continues to tap into, and evolves alongside, new developments in our fundamental understanding of relevant physical mechanisms and the predictability of those mechanisms. Thus, this review aims to summarize recent progress made not only in the field of initialized climate prediction using CGCMs but also in closely related areas such as potential predictability, mechanisms, and observed linkages.

## Foundations

### Mechanisms of Decadal to Multi-decadal Atlantic Climate Variability

The use of CGCMs to study the physical processes contributing to AMV is complicated by the large diversity of AMOC variability mechanisms, timescales, and climate impacts seen in different models. While some long coupled control simulations with current state-of-the-art CGCMs exhibit strong, regular multi-decadal AMOC oscillations (e.g., [50]), others show more broad-spectrum variability (e.g., [51]) or multiple distinct variability regimes within a single simulation (e.g., [52]). A variety of mechanisms have been invoked to explain intrinsic AMOC variability at different timescales in different models, and Buckley and Marshall have recently reviewed the copious literature on that topic [43•]. They group the low-frequency mechanisms into two broad categories: (1) those involving changes in North Atlantic deep convection (e.g., [53]) and (2) those involving baroclinic Rossby waves (e.g., [54]). Their review emphasizes that, regardless of the generative mechanism, decadal AMOC variability can be understood as essentially reflecting geostrophic dynamics dominated by western boundary buoyancy in the transition zone between the subtropical and subpolar gyres [55–57].

Since the early work of Delworth et al. [7], AMOC has repeatedly been identified as a driver of AMV in a multitude of CGCMs [3, 51, 58, 59]. The physical mechanism explaining the evolution of the surface signature of a positive AMOC anomaly—a warming in the subpolar gyre (SPG) and a cooling in the Gulf Stream (GS) region—involves southward propagation of AMOC anomalies emanating from high latitudes [60•]. Recent multi-model analyses bolster the case for the dominance of convection-related mechanisms, with consistent links found between high-latitude mixing, SPG densification, AMOC, and AMV in current-generation long coupled control simulations [61, 62]. The mean state bias in the subpolar Atlantic appears to dictate whether temperature or salinity dominates Labrador Sea density variations, and so the diversity of mean biases probably contributes to the inter-model diversity of feedback mechanisms and variability timescales [63•].

As with AMOC itself, the characteristics of AMV and the AMOC-AMV linkage vary from model to model, and external forcing complicates the relationship in ways that remain poorly understood [64–67]. Analysis of Coupled Model Intercomparison Project phase 5 (CMIP5) historical simulations reveals deficiencies in the amplitude and spatiotemporal evolution of simulated AMV, together with associated impacts, which raise questions about the fidelity of current models for studying Atlantic climate variability [68, 69]. Indeed, the precise role of slow, thermohaline ocean dynamics in generating AMV (and in particular, explaining the observed AMV) remains a topic of considerable ongoing debate (see discussion in Buckley and Marshall [43•] and references therein). For example, Häkkinen and coauthors argue that wind stress curl-forced changes in gyre strength account for recent decadal variations in SPG hydrography [27, 70, 71]. Clement et al. have also recently called into question the AMOC-AMV linkage by highlighting the similarities between AMV simulated with full CGCMs and configurations where an active ocean model is replaced with a slab ocean model [45]. However, an earlier study using the same methodology highlighted large differences in AMV spatial structure, amplitude, and atmospheric impacts [72]. Furthermore, recent papers [73•, 74, 75] have countered that low-frequency ocean forcing is critical for simulating realistic SST/heat flux relationships that underpin observed AMV climate impacts [29•].

Observations remain too limited to definitively settle the debate, but dominant patterns of subsurface ocean variability that appear to be distinctive signatures of AMOC variability support a role for AMOC in recent Atlantic decadal SST variability [76–79]. The RAPID-Meridional Overturning Circulation and Heat Flux Array (RAPID-MOCHA) at 26.5° N has now permitted connections to be established between interannual variations in AMOC, ocean heat transport, and near-surface ocean heat content in the subtropical Atlantic [80, 81•]. On multi-decadal timescales, long tide gauge records also support a causal relation between Atlantic ocean circulation change and observed twentieth century AMV [28•].

Ocean model simulations forced with historical surface fields from atmospheric reanalyses consistently show a strong AMOC intensification between the mid-1970s and the mid-1990s, followed by a weakening in more recent years [82•]. The simulated AMOC variability is in line with that inferred from observed subsurface temperature variations [77]. This slow AMOC spinup, that preceded the switch to positive AMV in the late 1990s [83], has been attributed to increasingly strong and persistent high-latitude buoyancy forcing associated with the observed positive trend in winter NAO [19, 79, 84, 85]. Atmospheric conditions over the SPG, and in particular over the Labrador Sea, exert a strong control on deep mixing, Labrador Sea Water (LSW) formation, deep ocean density, and AMOC/THC in realistic model simulations [85, 86]. Curiously, ocean reanalysis products that are constrained by subsurface observations show less agreement on the magnitude (or even the sign!) of historical decadal AMOC trends than corresponding surface-forced simulations [87•]. A pair of important recent studies by Delworth and colleagues uses a full CGCM (with more realistic air-sea coupling than in forced ocean simulations) to demonstrate in a controlled way how NAO heat flux forcing drives AMOC, AMV, and associated wider climate impacts [20•, 88••]. Their results imply that much of the observed low-frequency Atlantic climate variability of the last half-century is consistent with the NAO having a strong, delayed influence on AMOC and AMV.

The fact that buoyancy forcing accounts for most of the low-frequency variation in AMOC in the late twentieth century in reanalysis-forced ocean experiments [79, 84, 85] exemplifies why it is common to refer to either AMOC or THC interchangeably in the context of Atlantic decadal to multi-decadal variability. However, forced experiments reveal that the THC includes significant low-frequency changes in gyre strength (particularly, the SPG), and not just AMOC [89]. As low-frequency (buoyancy-forced) AMOC and gyre changes go hand in hand [90], with both circulation components contributing to heat transport variability [91], a conceptual refocus on THC instead of AMOC may be warranted in Atlantic variability and prediction work.

Recent advances in our understanding of how low-frequency, THC-related Atlantic Ocean heat content and SST variations impact other components of the Earth system have important implications for Atlantic climate prediction. Not only does AMOC appear to correlate with seasonal mean climate signals over land but also with (possibly) predictable shifts in surface climate extremes that have the greatest societal impact [92, 93]. Furthermore, AMOC-related heat transport through the Nordic Seas is a key predictor of simulated Arctic sea ice extent, and it appears to control low-frequency atmospheric heat transport variability in that region, in line with Bjerknes’ compensation hypothesis [4, 37•, 94, 95, 96•, 97•]. A key area of ongoing research that bridges mechanisms and impacts is determining whether AMV arises from a coupled ocean-atmosphere mode, with THC-driven SST variability driving NAO variability that potentially feeds back on THC and amplifies AMV [98]. Recent analyses of an extended atmospheric reanalysis dataset suggest that this is indeed the case; the observed AMV is anticorrelated with winter NAO conditions [30•, 31•, 83]. This observed relationship is also seen in some CGCMs, although it appears weak, and has been linked to AMOC-driven SST variability [35, 99, 100]. However, other studies find that the NAO response to AMV is primarily due to tropical SST forcing which may be only indirectly related to AMOC-driven heat convergence [101]. An outstanding research question is the role of stratospheric dynamics in this AMV-NAO linkage, with some studies suggesting that stratosphere-resolving models are key [102•] while others find an NAO response to AMV without using a high-top atmosphere [30•, 31•, 103]. Most current CGCMs do not incorporate stratosphere-resolving atmospheres, and this may be one of the factors contributing to an underrepresentation of coupled dynamics (along with other factors, such as low horizontal resolution, which we discuss below).

### Predictability of Decadal to Multi-decadal Atlantic Climate Variability

Several studies of long timescale climate predictability using comprehensive CGCMs published in the late 1990s and early 2000s contributed significantly to the advent of practical decadal climate prediction in the North Atlantic. The influential work of Griffies and Bryan examined ensembles initialized from different coupled model states to show that North Atlantic variability is potentially predictable for more than a decade in advance, with stronger and more regular THC oscillations conferring greater predictability [104]. They found clear decadal predictability for THC-related water mass variations (reflected in oceanic fields such as dynamic topography) but considerably shorter predictable timescales (of order a few years) for near-surface fields subject to high-frequency atmospheric noise. Similar “perfect model” predictability studies (both diagnostic and prognostic—see [105]) performed with a variety of different models consistently showed decadal-scale predictability of Atlantic THC variations. However, they exhibited much less agreement regarding surface climate predictability timescales: ranging from 1 year [106] to multi-decadal [107] (see references therein). Multi-model analyses highlighted the subpolar North Atlantic (SPNA) south of Greenland as a region of high and robust potential predictability, with SST/SAT predictability related to, but generally less than, MOC predictability [105, 108].

Perfect model predictability is derived from inevitably flawed representations of the Earth system, and there is substantial difference from model to model [105, 108–110]. Therefore, multi-model ensemble approaches likely offer the best prospects for robust conclusions about the real system, and such studies consistently show that the SPNA is a promising region for decadal prediction but with low signal-to-noise over land [105, 111, 112]. However, the assumption that perfect model predictability reflects an upper limit of real-world predictability, insofar as there is perfect knowledge of initial states and no drift, is called into question by recent initialized prediction results that show higher actual skill than would be expected from low signal-to-noise ratios [113••, 114•]. Whereas potential predictability studies focus on the predictable component of model variability (i.e., signal-to-noise), initialized experiments also permit an estimation of the predictable component of real-world variability (quantified as the correlation between observations and the forecast ensemble mean). A potential implication is that current models do not properly represent all of the mechanisms that give rise to real-world predictability in the North Atlantic sector and, as a result, are too “noisy.” However, more work is needed to understand why initialized prediction skill sometimes exceeds the potential predictability in real-world Atlantic prediction systems and to test the hypothesis that models respond too weakly to North Atlantic SST variability [115].

We are not aware of any studies showing that models with higher perfect model potential predictability achieve higher actual skill in initialized retrospective predictions, and therefore, the practical significance of Atlantic climate predictability diagnosed from particular CGCMs remains unclear. Boer et al. [116] do show that, for at least one model, there is a geographic correspondence between potential skill and actual skill for both the forced and internal components of SAT variability. Chapter 11 of the IPCC AR5 assessment report shows that the high potential predictability of internal SAT variations throughout the Northern Hemisphere extratropics in CMIP5 models (Fig. 11.1) corresponds to patterns of actual skill improvement in the Atlantic, but not in the Pacific (Fig. 11.4) [112]. Perhaps most importantly, such studies have helped identify important mechanisms and, in particular, the key role of ocean circulation change in Atlantic predictability [92, 104]. Recent perfect model studies have bolstered the consensus view that THC-related AMOC variability is predictable on roughly decadal timescales, that some initial states are significantly more predictable than others, and that enhanced AMOC predictability is closely related to enhanced predictability of AMOC-related heat content and surface climate fingerprints [110, 117–120].

## Initialized Decadal Climate Prediction

### The Subpolar North Atlantic and Nordic Seas

The high retrospective forecast skill in the North Atlantic seen in early initialized decadal predictions [47–49] helped to inspire a coordinated, international effort to advance decadal climate prediction as part of CMIP5 [121]. Most groups that participated in the CMIP5 protocol report modest improvement in global mean temperatures relative to uninitialized experiments but substantial improvement in the North Atlantic for up to a decade ahead [122–129, 130•]. This improvement appears to be largely insensitive to initialization method [131–134]. The subpolar North Atlantic (SPNA; comprising roughly the cyclonic ocean gyre north of about 50° N) is consistently the region with the largest relative improvement in surface temperature skill due to initialization, especially beyond the first few years of predictions [124, 130•, 131, 132, 135]. The improvement in skill is, in part, due to the initialization, and persistence, of substantial low-frequency variability of ocean heat content [136, 137]. However, several studies have shown significant improvements in the skill of upper ocean heat content and SST beyond persistence, especially for lead times longer than a few years [131, 136].

The historical time period over which initialized decadal predictions can be tested is strongly constrained by sparse observations (needed both for initialization and verification). In CMIP5, the earliest decadal hindcasts were initialized near 1960. The very limited sampling of observed decadal to multi-decadal variability makes skill assessment problematic. Therefore, there has been emphasis on understanding specific case studies of pronounced decadal change. The rapid warming of the SPG in the 1990s has been identified as a good test case for initialized predictions given its magnitude and occurrence in the relatively well-observed late twentieth century [138]. This warming has been found to be predictable in a number of independent systems, and the initialization of anomalously strong ocean heat transport has been identified as the key to their success [136–139, 140•]. Crucially, the initialization of anomalously strong ocean circulation, and in particular a strong AMOC, was found to play an important role, at least for the first few years of predictions. Although the initialization of anomalous upper ocean heat content (and associated advection of temperature anomalies) also plays a role [136, 137], the 1990s case study provides compelling evidence that large-scale ocean circulation anomalies are an important source of skill in decadal predictions of the late twentieth century.

**Fig. 1:**
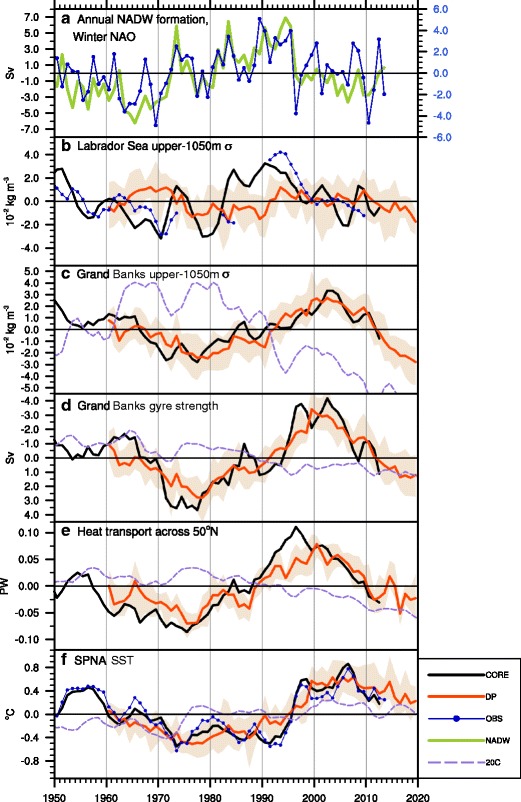
Modified from [96•]. **a** Annual rate of surface formation of North Atlantic Deep Water (NADW; σ_0_ > 27.6 kg m^−3^) over the high-latitude North Atlantic (60° W–20° E; 50° N–90° N) diagnosed from observed atmospheric and oceanic surface fields (*thick green curve*, *left axis)* and the observed winter (December–March) NAO index (*thin blue curve*, *right axis*). The remaining panels show 3-year running mean anomalies from a forced ocean-sea-ice simulation (CORE; *black curves*), CESM initialized decadal predictions averaged over the 5–7-year forecast period (DP; *red curves* and *shading* are ensemble mean and minimum/maximum range, respectively), CESM uninitialized twentieth century simulations (20C; *purple dashed curves* show the mean of a 6-member ensemble), and various observational time series (OBS; *blue curves*). Apart from the winter NAO in Fig. 1a, all time series are based on annual mean data. **b** Upper 1050 m density anomaly (σ_0_) in the central Labrador Sea region (56° W–49° W; 56° N–61° N). **c** Upper 1050 m density anomaly (σ_0_) in a region to the east of Grand Banks (50° W–35° W; 40° N–50° N). **d** Barotropic gyre streamfunction anomaly averaged over the Grand Banks region (note inverted axis; more negative values indicate stronger cyclonic circulation). **e** Ocean poleward heat transport across 50° N in the Atlantic. **f** SST in the subpolar North Atlantic (SPNA; 45° W–10° W; 50° N–60° N). See [96•] for further details

The evolution of hindcasts initialized in the early 1990s is broadly consistent with the idea of persistent positive NAO driving THC intensification in the late twentieth century [19, 79, 85, 88••, 139, 141]. The use of historical initial conditions imprints long-lasting NAO-driven density (and THC) anomalies into the coupled predictions [96•, 138, 140•]. However, although there is some skill in capturing multi-year THC-related overturning [142•] and gyre [96•] circulations, hindcasts do not appear able to capture the high-latitude formation of the deep density anomalies, and hence the onset of THC changes, in advance. It is important to underline that even if the hindcasts are not predicting changes in the ocean circulation per se, they may still reproduce the impact of the initialized anomalous ocean circulation and heat transport on the wider climate at decadal lead times [139]. Although the deep water formation processes in the Labrador Sea are not well-predicted, the southward propagation of pre-formed (i.e., initialized) water mass anomalies is highly predictable, and this propagation underpins the long lead time skill at predicting decadal, buoyancy-driven gyre fluctuations that modulate SPNA temperature [89, 96•] (Fig. 1).

Although there is general agreement that anomalously dense deep ocean conditions and a strong THC were key factors in the mid-1990s warming, other mechanisms were also at play. The negative NAO of 1995/1996 is also thought to have played a non-trivial role through reduced surface cooling and changes in surface currents [70, 79, 139]. However, the decadal prediction systems analyzed to date have not shown skill in predicting year-to-year variations in the NAO. The lack of skill in the NAO potentially explains why hindcasts do not generally capture the speed or magnitude of the observed warming and also why hindcasts tend to warm earlier than observed [136, 139]. In one study, ensemble members initialized in the early 1990s that, by chance, simulated a more realistic NAO did tend to exhibit a more realistic mid-1990s warming, but to first order a skillful NAO prediction was not necessary to predict the shift [139]. The mid-1990s warming has also been linked to a shrinking and weakening of the SPG [143–145]. Some systems have shown modest skill at predicting the SPG despite negligible skill at predicting the atmospheric forcing [146], suggesting the gyre changes may have been at least partly buoyancy-driven [89].

The propagation of THC-driven SPNA heat content anomalies into the Arctic via the Nordic Seas is considered a likely mechanism for decadal-scale predictability of Arctic upper ocean heat content, sea ice, and atmospheric heat flux [20•, 37•, 88••, 97•]. Yeager et al. argue that the mid-1990s warming of the SPNA (Fig. 1) contributed to the extreme rate of Atlantic sector winter sea ice loss that was observed between 1997 and 2007 and that the latter was predictable [96•]. Analysis of other systems reveals a wide range of skill in predicting SST in the Nordic and Barents seas, with the most skillful models showing indications of a heat content propagation mechanism at work [147].

Although much attention has focused on the prediction of the 1990s warming of the SPG, there are other events that have been studied. Several groups have shown that hindcasts can skillfully predict the cooling of the SPG in the 1960s [96•, 140•, 148, 149•] (Fig. 1) and even the warming of the North Atlantic in the 1920s [149•]. A detailed analysis of the SPG heat budget showed that skillful predictions of the 1960s cooling was, again, related to the initialization of ocean circulation and ocean heat transport (in this case anomalously weak) [148]. Analysis of ocean heat transport in other systems supports these conclusions [96•, 140•]. More recently, the SPNA upper ocean has again been cooling, which is also thought to be related to a slowdown in the THC [150•]. A continued near-term cooling of the SPNA has been forecast by a number of prediction systems, with implications for pan-Atlantic climate [96•, 140•, 151] (Fig. 1). Trying to predict these ongoing changes is a challenge that will test our understanding and modeling capabilities in near real time over the upcoming years.

### The Subtropical North Atlantic

Idealized model experiments suggest that tropical North Atlantic SST is the primary driver of global AMV teleconnections [21, 36•, 152, 153], and so skill in this region would seem to be necessary for foreknowledge of the most dominant and far-ranging AMV impacts. A handful of studies have highlighted an improvement of skill due to initialization, especially when averaging over lead times, in the subtropical North Atlantic (STNA) [130•, 154]. However, this improvement is often small in comparison to that seen in the SPNA [122, 125, 130•, 135]. The lower skill in the STNA is generally consistent with a more important role of the atmosphere in driving the changes in surface temperature in this region, given less ocean memory (i.e., shallower mixed layers) and weak advective heat convergence. In particular, changes in the strength of tropical winds and associated feedbacks, including the Wind-Evaporation-SST (WES) feedback and cloud-related feedbacks, are important drivers of STNA SST [45, 155, 156].

Model analysis suggests that extratropical temperatures can also drive the changes in the wind, by driving changes in the winter and summer NAO through increased baroclinicity or by driving changes in the Hadley cell, but these mechanisms are thought to be too weak in many models [135, 156, 157]. Finally, although the change in skill due to initialization in the STNA is relatively small, it is important to stress that overall skill in this region is positive in most CMIP5 models [125]. Whether the existing skill in this region is largely due to changes in greenhouse gases or other external forcing factors, such as anthropogenic or volcanic aerosols [158], remains to be understood in detail.

### Impacts Over Land

Although there is clear evidence of the positive impact of initialization on the North Atlantic Ocean, there is less convincing evidence of an impact on skill over land. Modest multi-year-lead skill improvement for surface temperature over the continents surrounding the Atlantic is present in some systems, particularly over Western Europe and North/Central America, but the improvement varies with season and seems most evident as a reduction of error rather than enhancement of correlation [123, 125, 127, 130•]. At least one study reports modest but significant skill in predicting temperature and precipitation climate extremes over North America and Europe at decadal lead times, although most of the skill appears to be due to external forcing rather than initialization [93]. There is also emerging evidence that rainfall over Africa can be predicted. Decadal prediction systems show robust skill improvement in capturing multi-year anomalies in the West African monsoon [159] and Sahel rainfall [160], with the source of the latter skill coming from AMV [161]. To circumvent poor sampling, some studies have employed a compositing technique that enhances signal-to-noise to show that significant changes in surface climate are simulated by predictions systems when anomalous temperatures are initialized in the North Atlantic [136, 137, 148, 149•, 162]. The changes identified as skillfully predicted include shifts in rainfall over North America, Africa and Northern Europe, and summer circulation over the North Atlantic, which are consistent with the expected impacts of anomalous Atlantic temperatures (e.g., [34]). Although evidence of skillful prediction over land is limited, the finding that decadal predictability may be underestimated in current decadal prediction systems is encouraging, as it implies that larger ensembles and/or improved models could yield improved skill scores over North America, Eurasia, and Africa in the future [114•].

Predicting Atlantic hurricane frequency at multi-year lead times is an exciting prospect with clear and tangible benefits to society, and several studies have suggested this may be a realizable goal [135, 140•, 163]. Skill improvement for tropical cyclone count is related to predictable changes in SPNA SST and, hence, the latitudinal temperature gradient in the North Atlantic and its impact on the Hadley cell [135, 157]. Most of the skill is related to the large shifts in the SPNA temperatures in the 1960s and 1990s, and there is some controversy over whether the skill is due to persistence of initialized SST or due to non-trivial prediction of SST changes [163, 164].

## Outstanding Questions and Future Prospects

Promising recent initialized prediction results (largely spanning ∼1960 to the present) support a strong role for NAO-driven THC anomalies controlling late twentieth century decadal variability in the SPNA. Furthermore, this high retrospective forecast skill in the SPNA would appear to explain the relatively high skill scores for SAT over the Western Europe and Scandinavia, and winter sea ice extent. However, the teleconnections (both oceanic and atmospheric) responsible for propagating skill from the SPNA to other regions remain poorly understood and probably poorly represented in current models. In addition, it is becoming clear that different regions of the North Atlantic (e.g., SPNA vs. STNA) are governed by distinct low-frequency mechanisms and, in turn, give rise to different impacts onto the wider climate system [36•, 101, 165]. This calls into question the usefulness of the traditional basin-wide AMV index for the purpose of attributing climate impacts, developing mechanistic understanding, and assessing and interpreting predictions.

The significance of other proposed mechanisms of low-frequency AMOC variability (e.g., baroclinic Rossby waves) remains to be demonstrated in the context of initialized Atlantic prediction, and it is an open (and perhaps unanswerable) question whether the skill of a prediction system is a strong function of the CGCM’s preferred AMOC mechanism. It is possible that, while the late twentieth century Atlantic was dominated by NAO-driven THC variability, other mechanisms may dominate in other time periods. New paleo-proxy reconstructions are critical for developing a deeper understanding of low-frequency Atlantic climate variability and associated mechanisms, such as the role of Nordic Seas overflows [166]. The extension of atmospheric reanalysis products backwards in time may permit more tests of initialized prediction over multiple AMV cycles, but there is large uncertainty in the ocean and sea ice state reconstructions generated from such products and little data to compare against [149•].

Most CMIP5-era prediction systems used rather simple initialization techniques, such as nudging to ocean state reanalyses or using ocean simulations forced with atmospheric reanalyses, and there is undoubtedly considerable room for improvement. Coupled data assimilation (DA) techniques offer the promise of high fidelity Earth system state estimates for initializing hindcasts in the modern observational era, and the same techniques could potentially be used to reconstruct much earlier ocean states if just SSTs are assimilated [167]. The potential to evaluate retrospective hindcasts over multiple AMV cycles makes the latter an appealing initialization strategy, but there is inevitably a trade-off between the length and quality of ocean state reconstructions. The pros and cons of alternative initialization methods, and the relative contributions to skill associated with initializing different Earth system components, are important topics of ongoing research that will help guide the development of future prediction systems.

An exciting prospect in Atlantic prediction work is the inclusion of prognostic biogeochemical models to facilitate forecasts of marine fields relevant to biology and the carbon cycle. New studies have appeared showing multi-year skill at predicting carbon uptake in the North Atlantic [168•] and net primary productivity in the tropical Pacific [169]. It remains to be seen whether AMV-related shifts in marine ecosystems [170] might also become achievable in future prediction systems.

### Towards Improved Model Fidelity

Model bias (used here to denote systematic errors in the representation of both the mean climate and its variability) is perhaps the single greatest impediment to improved decadal climate prediction. Hindcasts initialized from observed conditions (full-field initialization) drift towards the model’s preferred climatology, necessitating a drift-adjustment procedure prior to evaluation [116]. While such a posteriori corrections have been shown to yield skill scores comparable to anomaly initialization for select fields [133], key feedbacks between the ocean and atmosphere (e.g., ENSO development, cyclogenesis, or surface water mass formation) can be degraded by the presence of drift [171, 172]. The misrepresentation of the North Atlantic Current (NAC) path is a chronic bias in the non-eddy-resolving ocean models commonly used for decadal prediction, resulting in mean SSTs that are several degrees too cold in the extratropical North Atlantic. This bias impacts high-latitude air-sea exchange with important ramifications for AMV [173]. Model bias is a complex problem that can involve coupled processes that are notoriously difficult to unravel. While many coupled model biases in the Atlantic are probably related to poor Gulf Stream representation in coarse resolution ocean models, others, such as poor upper ocean thermal structure in the tropical Atlantic, appear to originate in the atmosphere [174].

Increased model resolution in the ocean and atmosphere is clearly a future frontier for decadal prediction research that will improve the physical realism of model systems and allow them to take maximum advantage of the modern observational network. Recent work has highlighted the role of mesoscale ocean fronts, and in particular Gulf Stream SST gradients, in driving the atmosphere—a mechanism that is largely absent in the non-eddy-resolving models that were used for CMIP5 predictions [175, 176]. Furthermore, high-frequency feedback between mesoscale ocean eddies and the atmospheric boundary layer appears to be key for realistic simulations of the dynamics and climate impacts of western boundary currents, requiring high horizontal resolution not only in the ocean but also in the atmosphere [177, 178]. The use of high-top, stratosphere-resolving models of the atmosphere is also expected to improve the realism of atmosphere-ocean coupling [102•]. While low-resolution prediction studies emerged from a rich literature on mechanisms, the nature of low-frequency Atlantic variability in the ocean-eddy-resolving regime remains largely unstudied given the tremendous resources required to run (and analyze) long simulations at such high resolution. Thus, there are many outstanding questions regarding the nature of underlying mechanisms at high resolution, how much of the knowledge gained through low-resolution studies will carry over, and whether North Atlantic predictability will be sensitive to resolution. To our knowledge, only one study has systematically assessed the change in decadal prediction skill associated with horizontal resolution [130•], and only one so far has looked at decadal predictability with an eddying ocean model [179]; more will undoubtedly follow.

Improved fidelity at simulating important North Atlantic air-sea interactions has recently been reported in the context of seasonal-to-interannual prediction. Using a relatively high-resolution model (stratosphere-resolving atmosphere at nominal 60 km resolution, and an ocean model at nominal 0.25°), the UK Met Office has demonstrated unprecedented skill at predicting NAO from a few months to a year in advance [113••, 180•]. These studies are part of an emerging literature suggesting that current models are systematically underestimating the potential predictability of the atmosphere in the North Atlantic and that large ensembles can be used to overcome excessive noise in the current generation of models. Which of several model improvements led to the improved predictions is not well understood, but given the important role played by the NAO in Atlantic decadal variability, this advancement could point the way for improved predictions on multi-annual to decadal timescales.

### The Role of External Forcing

There are many outstanding questions regarding the role that external forcings have played in shaping the real-world evolution of North Atlantic climate (e.g., [78, 158]), or that simulated in retrospective predictions (e.g., [122]), and this uncertainty hangs like a question mark over recent decadal prediction skill assessments. External forcings (i.e., prescribed time-varying radiative forcings associated with greenhouse gases and anthropogenic and natural aerosols) are undoubtedly a source of skill in the North Atlantic, especially in the tropical North Atlantic. This conclusion is based on potential predictability analyses (e.g., Fig. 11.1 of [112]) as well as joint analysis of initialized and uninitialized ensembles that share identical external forcing [125]. However, the number of ensemble members required to effectively isolate the forced signal from uninitialized simulations (in order to quantify the impacts of initialization) is not well constrained, and larger ensembles than have been used to date are likely required for robust statistics [116, 181]. Furthermore, the reasons why the forcings are a source of skill are not understood in detail. Changes in forcing could lead to improved predictions of SST by directly modulating the local surface heat budget, for example. Alternatively, the surface response to forcing may involve dynamical changes in the ocean and/or atmosphere. For example, recent studies suggest a lagged link between the solar cycle and the NAO [180•, 182]. Changes in volcanic and anthropogenic aerosols could also excite lagged NAO and/or AMOC variations [183, 184•]. It follows that Atlantic skill scores may be biased high due to the application of what would be unforeseeable volcanic aerosol loadings in retrospective predictions [185•]. Therefore, improving our representations of external forcing factors, our predictions of how they will change over the upcoming decade, and our understanding of model response to those forcings is critical for improving predictions and understanding the origin of skill.

## Conclusions

We have reviewed here some key recent developments in decadal prediction of the North Atlantic, but inevitably have left out mention of many relevant papers given the breadth of this topic. Although there have been important advances over the past decade, Atlantic climate prediction research has not seen any major paradigm shift away from the basic conceptual framework laid out in the review by Latif and coauthors in 2006 [44]. The slow flywheel of the Atlantic thermohaline circulation, set into motion by multiple consecutive winters of anomalous NAO buoyancy forcing, drives predictable surface temperature change in the North Atlantic on decadal timescales. A decade worth of new analysis of observations and models, including a greatly expanded set of initialized CGCM prediction simulations, has revealed the following:

- The subpolar North Atlantic (SPNA) consistently stands out as the most improved region in retrospective decadal predictions of upper ocean heat content and surface temperature in state-of-the-art initialized climate predictions (where improvement is assessed relative to externally forced simulations of the twentieth century that are not initialized from observed conditions). Many prediction systems show skill improvement here for up to a decade ahead, significantly outperforming persistence at long lead times.
- In some systems, high skill in the SPNA appears to reverberate around the Atlantic sector as improved skill in predicting surface climate over land in Europe, upper ocean heat content in the Nordic Seas and decadal Arctic winter sea ice trends, and Atlantic tropical cyclone frequency.
- The skillful prediction of late twentieth century and early twenty-first century SPNA variability is attributable to the initialization of (but not necessarily prediction of) NAO-driven water mass anomalies—and in particular, Labrador Sea Water anomalies. This sets up an anomalous thermohaline circulation (which includes both gyre and overturning components) with associated anomalous ocean heat transports. There are indications that the THC evolves somewhat predictably, due to the persistence and propagation of initialized, anomalous water masses, but there is a general lack of skill in predicting NAO and associated surface forcing.
- Surface temperature skill improvement in the tropical North Atlantic is less obvious and consistent than in the SPNA. There is skill overall, which suggests that external forcings are an important driver of variability in this region. However, more work is needed to understand the relative importance of various driving mechanisms, and related model shortcomings, in the tropical Atlantic.
- Recent work lends new support to the hypothesis that ocean may have an important influence on the extratropical atmosphere which could affect the magnitude and timescale of AMV. However, due to the short observational record and the likely influence of external forcing factors, the extent to which AMV represents a coupled ocean-atmosphere mode of variability remains an open question.

Initialized climate predictions of the North Atlantic have therefore begun to live up to the promise garnered from the past half-century of research. However, gaps in understanding continue to limit our confidence in predictions of future (rather than past) changes. Further progress will require a deeper understanding of Atlantic climate variability and relevant mechanisms, with AMV and its impacts still a primary area of focus. It is also clear that we need a deeper understanding of the behavior of initialized coupled prediction systems themselves with respect to a host of issues that we have not covered here in detail, such as drift, initialization shock, optimal ensemble size, ensemble generation, external forcing, and sensitivity to initial conditions. Systematic exploration of the sources of skill are illuminating (e.g., [186, 187]), as are process-oriented studies that help identify the mechanisms behind North Atlantic variability [36•, 88••] and skill. In addition to standard hindcasts with next-generation CGCMs, the Decadal Climate Prediction Project has called for several such targeted investigations in CMIP6 that should help spur advances for years to come [188]. Finally, the steady advancements seen in the field of numerical weather prediction over the last century give us good reason to expect similar progressive improvements in our ability to predict the Atlantic on interannual to decadal timescales as models and initialization techniques improve, as the observing system expands, and as computing power increases [189].
